# Performance of the WHO 2011 TB Symptom Screening Algorithm for Pulmonary TB Diagnosis among HIV-Infected Patients in Gondar University Referral Hospital, Ethiopia

**DOI:** 10.1155/2016/9058109

**Published:** 2016-12-12

**Authors:** Martha Alemayehu Menberu

**Affiliations:** Department of Medical Microbiology, School of Biomedical and Laboratory Sciences, University of Gondar, Gondar, Ethiopia

## Abstract

The new WHO 2011 guidelines on TB screening among HIV-infected individuals recommend screening using four TB symptoms (current cough, fever, weight loss, and night sweats). This study aimed to assess the performance of WHO 2011 TB symptom screening algorithm for diagnosing pulmonary TB in HIV patients and identify possible risk factors for TB. Institutional based cross-sectional study was conducted from February 2012 to November 2012. A total of 250 HIV-infected patients aged ≥18 years visiting the University of Gondar Hospital, ART clinic, were enrolled. Information about WHO TB clinical symptoms and other known risk factors for TB was collected using structured questionnaire. Spot-morning-spot sputum samples were collected and direct AFB microscopy, sputum culture, and RD9 molecular typing were performed. Statistical data analysis was performed using SPSS Version 20.0 software. Of 250 study participants, fever was reported in 169 (67.6%), whereas cough and night sweats were reported in 167 (66.8%) and 152 (60.8%), respectively. A total of 11 (4.4%) TB cases were identified. Of these, 82% (9/11) TB patients reported cough, so that the negative predictive value was 98%. In addition, 66% (158/239) TB negative patients reported cough, so that positive predictive value of cough was 5%. According to the new WHOTB symptom screening algorithm, out of 250 HIV-infected persons, 83% (5/6) have been investigated by TB symptom screening and AFB smear microscopy. Therefore, the 2011 WHO TB symptom screening tool for the diagnosis of pulmonary TB is likely to reduce the diagnostic delay and lower TB morbidity and mortality rate particularly in HIV prevalent settings.

## 1. Introduction

Tuberculosis is an infectious disease that continues to be major public health problem in Ethiopia due to the expansion of HIV epidemic [1]. Increased disease burden caused by TB and HIV coinfection makes it critically essential to accurately detect TB infection among persons with HIV [2].

World Health Organization (WHO) has recommended screening of active TB in all HIV positive patients, with therapy provided to active TB infection or isoniazid preventive treatment for latent TB in order to reduce mortality [3]. However, as of 2010, among 33.3 million individuals with HIV, only 2.3 million have been checked for TB infection, and of those without active TB 178,000 were offered preventive therapy [1]. The lack of TB screening is due in large part to the limitations of currently used screening tests.

Different studies have shown that the existing clinical signs and symptoms of TB in HIV-infected individuals vary from those of people who are HIV negative; for example, many HIV-infected and culture positive TB patients are not showing cough, which is one of the common TB symptom screening questions for TB control programs [4]. Ethiopian guidelines suggest that TB screening should be performed in HIV patients by combining TB symptom screening with that of sputum AFB smear microscopy and chest x-ray [2]. However, only limited numbers of HIV-infected individuals are checked for TB due to limited resources and lack of well formulated national implementation guideline.

Studies have suggested that smear AFB microscopy and chest x-ray, which are the common accessible TB screening tools, may not be sufficient screening tools for HIV coinfected active TB patients. There are also increased smear negative and extra pulmonary TB among HIV positive individuals, which can make the diagnosis of TB challenging [5]. Sputum culture can increase TB positivity among HIV-infected individuals, but does not always exist in resource limited areas [2].

The new WHO 2011 tools for screening of TB and IPT in HIV positive people were approved and employed in Cambodia and South Africa by the year 2010. The tools recommend symptom screening of current cough, fever, weight loss, and night sweats and giving IPT when these symptoms are not present [6]. The negative predictive value of this screening tool in those HIV positive people with 5% TB prevalent setting is 97.7% [7]. The progress of active TB in HIV-infected individuals depends on immune status, high-risk condition, and way of life and contact history with active TB patients [8–10]. Therefore, this study aimed to assess the performance of 2011 WHO TB symptom screening algorithm for diagnosing pulmonary TB and to identify potential risk factors for the existence of pulmonary TB in HIV positive individuals at the referral hospital of Gondar, Ethiopia.

## 2. Materials and Methods

### 2.1. Study Design, Period, and Area

Institutional based cross-sectional study was conducted from February 2012 to November 2012 at the University of Gondar Teaching and Referral Hospital among HIV/AIDS adults attending ART clinic. The referral hospital provides care as well as treatment service for TB and HIV patients. Currently there are about 9,470 HIV patients on follow-up in ART department of Gondar; out of these 6,000 patients are starting ART and 3,470 patients are on pre-ART.

The sample size was determined by single population proportion method, assuming asymptomatic TB disease prevalence of 16% in newly diagnosed HIV patients based on Shah et al., 2009, in Addis Ababa, Ethiopia [2], and considering 95% level of confidence, 5% margin of error, and 10% contingency. Therefore, a total of 250 HIV patients were enrolled consecutively.

HIV-infected patients aged ≥18 years visiting the University of Gondar Hospital, ART clinic, and who have any of TB symptoms including cough, fever, weight loss, and night sweats at the time of study were included. Sociodemographic and clinical data including TB symptoms were collected for each study patient using a structured questionnaire to evaluate the performance of WHO 2011 TB screening algorithm for diagnosing TB among HIV patients. In addition, known risk factors for TB including housing, alcohol drinking habit, previous contact with individuals with active TB, and employment history were also collected by a medical doctor and nurses via interview during the patient visit.

### 2.2. Inclusion and Exclusion Criteria

HIV positive individuals aged ≥18 years who agreed to participate and provided consent and who were on Pre ART or are currently receiving ART and those who had previous TB history were included. Those HIV positive individuals with severe disease unable to give sputum specimen were excluded from the study.

The spot-morning-spot sputum specimens were collected from eligible HIV patients to this study by the laboratory technologists at the University of Gondar Hospital ART laboratory. A direct AFB smear microscopy was performed using the conventional Ziehl-Neelsen staining technique at the school of biomedical & laboratory sciences research laboratory (Biomedical Complex Laboratory), and then, the three sputum samples were pooled together in 20 ml sterile screw capped universal test tubes and kept in deep freeze at −20°C and all sputum samples were transported in ice box (at +4°C) to the core research laboratory at Armauer Hansen Research Institute (AHRI), Addis Ababa, for sputum culture.

The sputum samples were decontaminated and homogenized by the modified Petroff's method. About 1 ml of the sediment was inoculated into the conventional LJ medium with 0.6% sodium pyruvate and glycerol for primary isolation. After inoculation, LJ slants were held for 8 weeks at 37°C and were examined visually for bacterial development and growth every day in the first week and then twice per week thereafter for the total of 8 weeks for the presence of mycobacterial colonies.

### 2.3. Operational Definitions

*Smear Positive Pulmonary Tuberculosis*. It means patients who are AFB positive with two initial sputum smear microscopy examinations.

*Pulmonary Tuberculosis*. Tuberculosis typically affects the lungs.

*Extrapulmonary Tuberculosis*. Presence of TB illness outside of the lung parenchyma.

### 2.4. Data Analysis

Statistical data analysis was carried out by SPSS software packages (Version 20.0). The sensitivity and specificity, as well as predictive value of symptoms, were calculated using presence of individual symptoms and sputum culture as a gold standard method. Analyses of univariate and multivariate logistic regression were also performed to identify potential risk factors associated with the development of pulmonary TB and *P* value < 0.05 was regarded as statistically significant association.

### 2.5. Ethical Consideration

Ethical approval was gained from the ethics committee of University of Gondar, School of Biomedical and Laboratory Sciences, and AHRI/ALERT ethical clearance board. Written consent was also obtained from the study subjects. Confidentiality was guaranteed through coding during data documentation.

## 3. Results

### 3.1. Sociodemographic Characteristics

A total of 250 HIV positive patients were diagnosed for TB. The study participants comprised 92 (36.8%) males and 158 (63.2%) females with 18 to 70 years of age range. In addition, the mean age of study subjects was 35.72 years with a standard deviation of 9.42 (Table 1). Of the total participants, 44% were married and percentage of urban versus rural resulted as 84% whereas housewives and unemployed comprised 18% and 54.4%, respectively (Table 1).

### 3.2. TB Symptom Distribution

**Figure 1:**
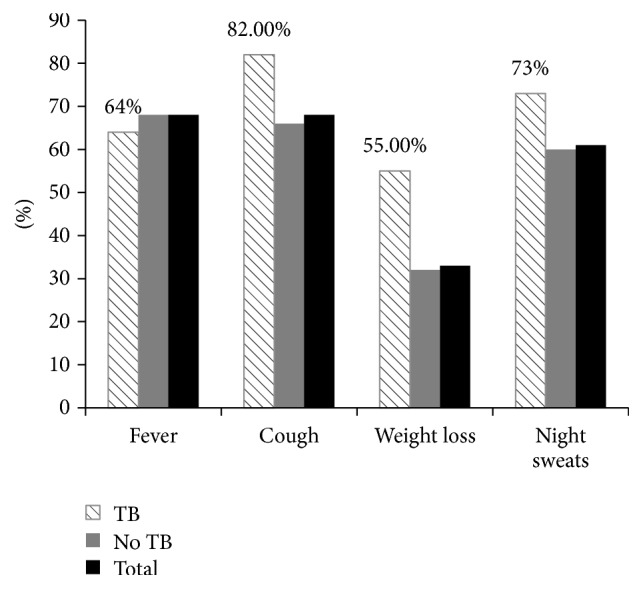
TB symptom distribution of the study participants in Gondar, North West Ethiopia, 2012.

Of 250 study participants, fever was reported in 169 (67.6%) of the study participants, whereas cough and night sweats were reported in 167 (66.8%) and 152 (60.8%) of the study participants, respectively. From all participants, a total of 11 (4.4%) TB cases were identified using smear microscopy, culture, and molecular RD9 typing test. Out of eleven TB positive participants 9 (82%) had cough and 8 (73%) had night sweats (Figure 1).

### 3.3. The Performance Characteristics of Individual Clinical Symptoms

The sensitivity, specificity, and the predictive value of the four TB symptoms, as screened individually, are indicated in Table 2. Cough of different extent had 82% sensitivity, so that the negative predictive value was 98%. On the other hand, 66% (158/239) TB negative patients reported cough; therefore the positive predictive value was 5%. Individual TB symptom screening for fever with different duration would identify 64% TB patients, and weight loss and night sweats would identify 55% and 73%, respectively. The specificity of fever, weight loss, and night sweats was 32%, 68%, and 40%, with positive predictive value of 4%, 7%, and 5%, respectively. The negative predictive values for individual TB symptom screenings were more than 95% and the result indicated fever (95%), weight loss (97%), and night sweats (97%). Therefore, presence of individual clinical symptoms was used to identify pulmonary TB cases and could reduce the diagnostic delay of TB patients.

According to the new WHO TB symptom screening algorithm, out of 250 HIV positive persons included in this study, 83% (5/6) would be identified by TB symptom screening and sputum direct smear microscopy. In this study, additionally, 100% (6/6) of TB cases were identified using TB symptom screening and culture method. Therefore, the new WHO TB symptom screening algorithm detects 83% of study methods that minimizes TB morbidity and mortality among HIV prevalent areas. On the other hand, none of the variables assessed in this study showed significant association (Table 3).

## 4. Discussion

Tuberculosis is the major opportunistic infection and principal cause of mortality among HIV positive individuals. Moreover, TB diagnosis among persons with HIV is a dominant public health problem. The WHO symptom screening algorithm for the detection of pulmonary TB among HIV positive individuals needed confirmation particularly in settings with limited resources to implement the guidelines. This brings method difficulties because it is challenging to build a good research environment in these circumstances allowing for the correct assessment of diagnostic accuracy of the guideline. This study evaluated the performance of the WHO 2011 algorithm for detection of pulmonary TB among HIV positive individuals and also tried to assess the possible associated risk factors of TB in HIV positive individuals.

In the present study, cough with different duration had 82% sensitivity, 34% specificity, and also the negative predictive value of 98%. The latest meta-analysis study aimed to build up standardized TB screening algorithm for HIV positive individuals and showed 78.9% sensitivity and 49.6% specificity [7]. However, it was well-known that the negative predictive value of TB screening algorithm depends on TB prevalence rate in screened population, ranging from 97.7% in TB prevalence of 5% to 90% in TB prevalence rate of 20%. This finding is in line with our result in that routinely available TB screening tests have limited sensitivity for detecting active TB disease among HIV-infected persons.

In addition, the 4.4% rate in our study is similar to that found in other studies evaluating TB screening algorithms among HIV-infected persons [11]. Nonetheless, HIV care and treatment settings must consider TB disease prevalence when estimating the likely performance of any screening algorithm.

Our study identified 4.4% (11/250) cases of undiagnosed pulmonary TB among HIV-infected individuals but according to the new WHO TB symptom screening algorithm, out of 250 HIV-infected persons, 83% (5/6) would be identified by TB symptom screening. Even though TB symptom screening clinical tool will ignore 4.4% of HIV positive individuals with undiagnosed TB, in mainly high-burden minute resource areas, this algorithm will be adequate to rule out active TB.

Even though TB symptom screening is unsuccessful in detecting all TB patients with culture positive, our findings indicated that it can act as valuable means for identification of active TB; patients without cough, fever, and night sweats are not likely to develop TB disease (specificity of 68%, negative predictive value of 97%). Therefore, TB symptom screening could be a valuable tool for follow-up and treatment purposes of HIV positive individuals to apply for ruling out active TB among individuals being regarded for ARV treatment or IPT.

In the present study, sputum smear microscopy identified few (4.4%) cases of pulmonary tuberculosis among patients who did not show cough >2 weeks. This result is consistent with another study conducted by Cain et al. [12], which showed that extra sensitive screening approach for diagnosis of PTB by combination of symptoms as admission for PTB [4, 7, 13, 14] without identifying the cough period would possibly end up in the PTB path.

An approach that joins TB symptom screening with AFB smear microscopy and chest x-ray (e.g., recent guidelines in Ethiopia) would distinguish 62% TB patients, with 82% specificity and elevated negative predictive value. Due to lack of widespread accessibility of TB culture, the 3-step approach may present a suitable stability as the tool misses the smallest number of TB patients, while reducing overidentification and therapy [2].

In the current study, sputum culture resulted identification of additional TB cases but the method is time taking. A study conducted in South Africa showed, the Gene Xpert MTB/RIF assay increased TB case detection rate by 45% [15]. Therefore, we need a diagnostic method with a minimum turnaround time than culture.

In the present study, different risk factors were evaluated for their association with the occurrence of TB disease. The results showed that the occurrence of TB disease and the frequency of the isolates of* Mycobacterium* isolated from TB cases were not affected either by age, sex, residence area, occupation, marital status, educational status, alcohol intake, previous contact with tuberculosis patients, and TB symptoms in PTB (*P* > 0.05). Our result is consistent with other observations made by Alemie and Gebreselassie, 2014 [16], and Iwnetu et al., 2009 [17]. This might be due to the fact that all patients in this study who had history of TB infection did complete the full dose of antituberculosis treatment and were declared as cured cases.

The limitations of this study included the following. Firstly the algorithm performance at various levels of TB prevalence could not be assessed. Secondly, we were incapable of comparing the performance of the new with that of the old WHO algorithm. Thirdly, patients without symptoms were not included so we could not compare the performance of the guideline with those patients with TB symptom. In addition, we applied solid culture as standard for identification of pulmonary TB. Culturing various samples on liquid media would have identified further TB confirmed patients [18, 19] and affected the sensitivity and specificity of diagnostic method in HIV-infected TB suspected individuals.

## 5. Conclusions

The diagnostic accuracy of the new WHO TB symptom screening tool to diagnose pulmonary TB is good and minimizes TB morbidity and mortality among HIV patients. The guideline has also implications for conclusions to start tuberculosis preventive treatment for patients in the ART service.

## Figures and Tables

**Table 1:** Sociodemographic characteristic features of the study participants in Gondar, North West Ethiopia, 2012.

Sociodemographic characteristics	Frequency	Percentage
*Sex*		
Male	92	36.8
Female	158	63.2
*Age, mean (SD)*	35.72 (9.42)	
*Residence area*		
Urban	210	84.0
Rural	40	16.0
*Marital status*		
Single	47	18.8
Married	111	44.4
Divorced	52	20.8
Widowed	40	16.0
*Educational background*		
Illiterate	98	39.2
Read & write	135	54.0
Higher education	17	6.8
*Occupational status*		
Employed	38	15.2
Unemployed	136	54.4
Retired	4	1.6
Housewife	45	18.0
Daily laborer	20	8.0
Farmer	7	2.8

**Table 2:** Predicting pulmonary tuberculosis based on the existence of individual clinical symptoms in Gondar, North West Ethiopia, 2012.

Symptom(s)	Sensitivity	Specificity	PPV	NPV
	(%)	(%)	(%)	(%)
Fever	64	32	4	95
Cough	82	34	5	98
Weight loss	55	68	7	97
Night sweats	73	40	5	97

**Table 3:** Risk factors for TB in HIV positive individuals in Gondar, North West Ethiopia, 2012.

Characteristic	Univariate analysis		Multivariate analysis	
	OR (95% CI)	*P*	OR (95% CI)	*P*
Age (year)	1.02 (0.96–1.08)	0.60		
Female	1.58 (0.41–6.20)	0.51		
Married	0.27 (0.06–1.25)	0.09	0.31 (0.06–1.52)	0.15
Unemployed	0.69 (0.20–2.31)	0.54		
Illiterate	0.57 (0.15–2.20)	0.41		
Any alcohol use	1.75 (0.36–8.50)	0.49		
Previous contact with TB	1.17 (0.14–9.51)	0.89		
Household members ≥4	1.58 (0.47–5.34)	0.46		
Symptoms				
Fever	0.83 (0.24–2.93)	0.77		
Cough	2.31 (0.49–10.92)	0.29		
Weight loss	2.62 (0.78–8.87)	0.12	2.38 (0.67–8.43)	0.10
Night sweats	1.76 (0.46–6.80)	0.41		
